# Molecular characterization of vernalization and response genes in bread wheat from the Yellow and Huai Valley of China

**DOI:** 10.1186/1471-2229-13-199

**Published:** 2013-12-05

**Authors:** Feng Chen, Manxia Gao, Jianghua Zhang, Aihui Zuo, Xiaoli Shang, Dangqun Cui

**Affiliations:** 1 Agronomy College, Henan Agricultural University, 95 Wenhua Road, Zhengzhou 450002, China; 2 Key Laboratory of Physiological Ecology and Genetic Improvement of Food Crops in Henan Province, Zhengzhou 450002, China; 3 Collaborative Innovation Center of Henan Grain Crops, Zhengzhou 450002, China

**Keywords:** Bread wheat, Vernalization response genes, Photoperiod gene, Flowering days, Heading days

## Abstract

**Background:**

Flowering time greatly influences the adaptation of wheat cultivars to diverse environmental conditions and is mainly controlled by vernalization and photoperiod genes. In wheat cultivars from the Yellow and Huai Valleys, which represent 60%-70% of the total wheat production in China, the large-scale genotyping of wheat germplasms has not yet been performed in terms of vernalization and photoperiod response alleles, limiting the use of Chinese wheat germplasms to a certain extent.

**Results:**

In this study, 173 winter wheat cultivars and 51 spring wheat cultivars from China were used to identify allelic variations of vernalization and photoperiod genes as well as copy number variations of *Ppd-B1* and *Vrn-A1*. Two new co-dominant markers were developed in order to more precisely examine *Vrn-A1b*, *Vrn-B1a*, and *Vrn-B1b*. Two novel alleles at the *Vrn-B3* locus were discovered and were designated *Vrn-B3b* and *Vrn-B3c. Vrn-B3b* had an 890-bp insertion in the promoter region of the recessive *vrn-B3* allele, and *Vrn-B3c* allele had 2 deletions (a 20-bp deletion and a 4-bp deletion) in the promoter region of the dominant *Vrn-B3a* allele. Cultivar Hemai 26 lacked the *Vrn-A1* gene. RT-PCR indicated that the 890-bp insertion in the *Vrn-B3b* allele significantly reduced the transcription of the *Vrn-B3* gene. Cultivars Chadianhong with the *Vrn-B3b* allele and Hemai 26 with a *Vrn-A1*-null allele possessed relatively later heading and flowering times compared to those of Yanzhan 4110, which harbored recessive *vrn-B3* and *vrn-A1* alleles. Through identification of photoperiod genes, 2 new polymorphism combinations were found in 6 winter wheat cultivars and were designated *Hapl-VII* and *Hapl-VIII*, respectively. Distribution of the vernalization and photoperiod genes indicated that all recessive alleles at the 4 vernalization response loci, truncated “Chinese Spring” *Ppd-B1* allele at *Ppd-B1* locus and *Hapl-I* at the *Ppd-D1* locus were predominant in Chinese winter wheat cultivars.

**Conclusion:**

This study illustrated the distribution of vernalization and photoperiod genes and identified 2 new *Vrn-B3* alleles, 1 *Vrn-A1*-null allele, and two new *Ppd-D1* polymorphism combinations, using developed functional markers. Results of this study have the potential to provide useful information for screening relatively superior wheat cultivars for better adaptability and maturity.

## Background

The grain yield of wheat is determined not only by the genes directly controlling yield and yield components, but also by the genes controlling plant development and maturity [1]. Vernalization and photoperiod responses, which determine flowering and heading times, have a significant influence on the adaptability of wheat plants to a set of environmental conditions. The vernalization requirement of winter wheat cultivars (a prolonged exposure to low temperatures to accelerate flowering) protects the sensitive floral meristems from frost damage by cold temperatures. Differences in photoperiod sensitivity are also widely used in wheat breeding to provide adaptation to diverse agronomic environments.

Major vernalization response (*Vrn*) loci, which determine flowering and maturity times, have been mapped to the middle of the long arms of chromosomes 5 [2-5]. Moreover, vernalization response is actually controlled by 3 distinct *Vrn* loci in bread wheat. *Vrn-1* genes, directly influencing flowering and maturity times, are located on chromosomes 5AL, 5BL and 5DL [6,7] and were the first vernalization genes cloned in polyploid wheat by map-based cloning techniques [8]. Subsequently, *Vrn-2* and *Vrn-3* genes were cloned in wheat and barley by map-based cloning [9,10]. However, *Vrn-2* affected wheat growth habit as an indirect repressor of expression level of *Vrn-A1* by repressing *Vrn-3*[11]. Loss-of-function *Vrn-2* natural mutations or deletions result in the spring growth of bread wheat, which does not require vernalization to flower. The *Vrn-3* gene, a homolog of the *Arabidopsis FT* gene [10,12], exhibits increased expression if its dominant allele is present, resulting in accelerated flowering and a bypass of the vernalization requirement [10]. Therefore, the growth habits and vernalization requirements of cereal plants are mainly determined by 3 genes *Vrn-1*, *Vrn-2* and *Vrn-3*.

Photoperiod response is another important factor that influences the flowering and maturity of wheat plants. Photoperiod insensitivity is widespread in bread wheat production areas globally and is particularly prevalent in regions where the crop is grown during short days or where crop maturity is required before the onset of high summer temperatures [13]. Based on several previous reports [14-16], photoperiod response in bread wheat is mainly controlled by the *Ppd-1* loci on the short arms of chromosomes 2D, 2B, and 2A. The *Ppd-D1* allele for photoperiod insensitivity is generally considered the most potent, followed by *Ppd-B1* and *Ppd-A1*[17,18], though this view is still controversial due to conflicting results showing that *Ppd-B1a* could be as strong as *Ppd-D1*[19].

However, in the same type of wheat cultivars with winter or spring growth habits, there were still several-day differences among heading and flowering times. This difference mainly resulted from allelic variation in vernalization and photoperiod response genes at one or more loci. Therefore, identification of the vernalization and photoperiod response alleles will enhance the selection of cultivars with wide adaptability to a set of environments. Several vernalization and photoperiod response alleles have been identified in polyploidy wheat cultivars from different countries, subsequently leading to the development of a series of molecular markers for improved efficiency in identifying different vernalization and photoperiod response alleles [9,15,20-23]. Fu et al. [20] indicated that the Argentine and Californian spring wheat cultivars showed a lower frequency of the dominant *Vrn-A1* allele and a higher frequency of the dominant *Vrn-D1* allele relative to the worldwide collection, though the dominant *Vrn-A1* allele was the most popular genotype at the *Vrn-A1* locus. Iqbal et al. [21] developed new molecular marker for identifying *Vrn-A1* alleles and found that the dominant *Vrn-A1a* allele was the most prevalent while the dominant *Vrn-D1* allele was absent in the Canadian spring wheat surveyed. Zhang et al. [24] found that the dominant *Vrn-D1* allele showed the highest frequency in Chinese popular wheat cultivars (37.8%), followed by the dominant *Vrn-A1*, *Vrn-B1*, and *Vrn-B3* alleles. They also showed that the *Vrn-D1* allele is associated with the latest heading time, while *Vrn-A1* is associated with the earliest heading time and *Vrn-B1* is associated with an intermediate heading time. Santra et al. [22] and Shcherban et al. [25] identified 2 new *Vrn-B1* alleles and found that a majority of the wheat germplasm surveyed carried the dominant allele *Vrn-A1a* alone or in combination with *Vrn-B1*, *Vrn-D1*, or *Vrn-B3* alleles and that *Vrn-B1* and *Vrn-D1* alleles were almost always associated with other dominant *Vrn-1* allele(s). Golovnina et al. [26] characterized great nucleotide variability in the region from -62 to -221 of the *Vrn-1* promoter region in wild and cultivated wheats. Beales et al. [15] and Guo et al. [23] recently identified several polymorphisms in *Ppd-D1* and *Ppd-A1* loci of bread wheat.

Bread wheat (*Triticum aestivum* L.) is one of the 3 most important food crops in China, with more than 100 million tons produced annually in recent years. As a secondary origin center for bread wheat with a broad diversity of the germplasm, China is the largest producer and consumer of wheat in the world, and Chinese wheat germplasms differ from those of other countries in several aspects. Chinese wheat is mainly planted in 10 agro-ecological zones that are further divided into 26 sub-zones, with winter, facultative, and spring wheats sown both in autumn and spring [27]. Winter wheat occupies more than 85% of the total area and production of Chinese wheat. Of all agro-ecological zones, the Yellow and Huai wheat production region, covering all of Henan and parts of Shandong, Hebei, Shanxi, Shaanxi, Anhui and Jiangsu Provinces, is the most important and largest wheat production zone with 60%-70% of both total harvested area and total wheat production. In terms of requirements of temperature and days for vernalization, winter wheat cultivars from the Yellow and Huai Valley could be further divided into springness, semiwinterness and winterness wheat cultivars and are early in maturing in order to suit the double cropping system.

In this study we identified the distribution of vernalization and photoperiod response genes in winter wheat cultivars from the Yellow and Huai Valley, especially for landrace and current popular cultivars. In 2 landrace cultivars with winter growth habits, we found 2 novel *Vrn-B3* alleles, 1 *Vrn-A1*-null allele and 2 new polymorphism combinations of photoperiod response alleles. These results provide useful information for the utilization of Chinese wheat germplasms in terms of growth habits.

## Results

### Discovery of 2 novel dominant *Vrn-B3* alleles in Chinese winter wheat

**Figure 1:**
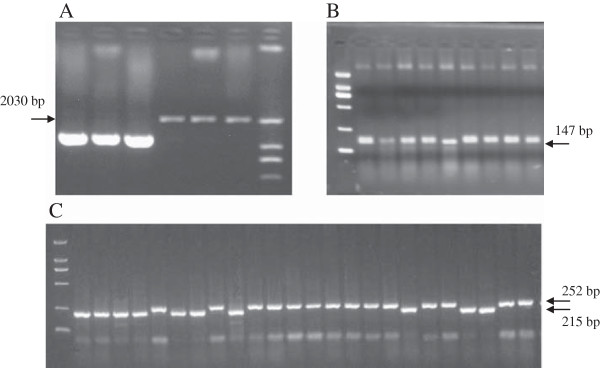
**Identification of vernalization response alleles by newly developed co-dominant markers in Chinese wheat cultivars. A**: Primer set Vrn-10 for identification of *Vrn-B3c* allele with 2030 bp. From left to right: Yunong 202, Yunong 201, Zhoumai 18, Chadianhong with three lanes, DNA ladder DL 2000. **B**: Primer set Vrn-2 for identification of *Vrn-A1b* allele with 147 bp. From left to right: DNA ladder DL 2000, Yumai 57, Aikang 58, Yunong 202, Jimai 38, Wuyimai, Huaimai 19, Bonong 5, Yumai 41, Yumai 51. **C**: Primer set Vrn-7 for identification of *Vrn-B1b* allele with 215 bp. From left to right: DNA ladder DL 2000, Gaoyuan 932, Gaoyuan 314, Gaoyuan 448, Gaoyuan 115, Qingchun 587, Qingchun 891, Qingchun 952, Gaoyuan 363, Gaoyuan 028, Gaoyuan 356, Gaoyuan 175, Gaoyuan 182, Gaoyuan 913, Humai 11, Humai 13, Humai 14, Humai 15, Minhe 588, Minhe 665, Lemai 5, Gaoyuan 602, Xinzhe 9, Qingchun 415, Qingchun 570.

**Figure 2:**
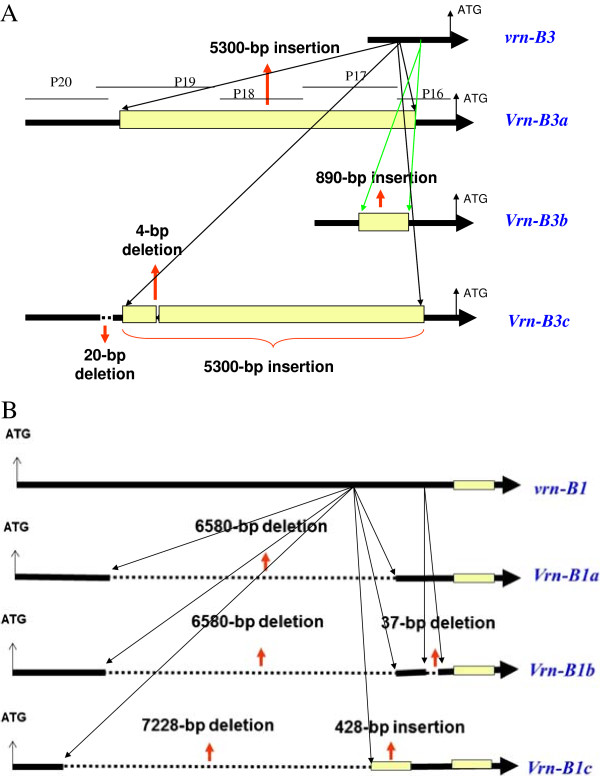
**Schematic representation of *Vrn-B3 *alleles identified in Chinese winter wheat cultivars. (A)** P16-P18 are primers we designated in this study for identification of 5300-bp insertion in promoter sequence of *Vrn-B3* gene) and *Vrn-B1***(B)** alleles identified in Chinese winter wheat.

Screening of the 173 Chinese winter wheat cultivars by dominant PCR primer sets Vrn-P12F/R and Vrn-P13F/R (Table 1) indicated that 170 cultivars with the expected 1140-bp fragment size belonged to the recessive *vrn-B3* allele. However, one Chinese landrace cultivar Chadianhong showed a fragment of approximately 2000 bp when amplified with primer set Vrn-P12F/R by 3 independent PCRs (Figure 1A), indicating that there was an approximately 900-bp insertion in Chadianhong in comparison with the recessive *vrn-B3* allele. The approximately 2000-bp fragment was ligated into the pGEM-T Easy vector, and sequencing results of plasmids containing the targeted fragment indicated that an exact 890-bp fragment was inserted into the 5′ untranslated region (UTR) at -429 bp (reference to ATG; Figure 2A and Table 2). This new *Vrn-3* allele was designated as *Vrn-B3b* (submitted to NCBI No: JN627519), according to the nomenclature of vernalization response genes by Fu et al. [20] and Yan et al. [9,10].

**Table 1:** **PCR primers for detecting vernalization response and *Ppd-D1 *** **alleles in bread wheat**

**Name**	**Allele or haplotype**	**Forward primer**	**Reverse primer**	**Expected band size (bp)**	**Annealing temp. °C**	**Reference**
Vrn_P1	*vrn-A1/Vrn-A1a/Vrn-A1b/Vrn-A1c*	GAAAGGAAAAATTCTGCTCG	TGCACCTTCCC(C/G)CGCCCCAT	950 + 876 or 714 or 734	50	[9]
Vrn_P2	*Vrn-A1b*	CCTGCCGGAATCCTCGTTTT	CTACGCCCCTACCCTCCAACA	147 or 167	63	In this paper
Vrn-P3	*Vrn-A1c*	AGCCTCCACGGTTTGAAAGTAA	AAGTAAGACAACACGAATGTGAGA	1170	65	[20]
Vrn-P4	*vrn-A1*	GCACTCCTAACCCACTAACC	TCATCCATCATCAAGGCAAA	1068	59	[20]
Vrn-P5	*Vrn-B1a*	CAAGTGGAACGGTTAGGACA	CTCATGCCAAAAATTGAAGATGA	709	63	[20]
Vrn-P6	*vrn-B1*	CAAGTGGAACGGTTAGGACA	CAAATGAAAAGGAATGAGAGCA	1149	58	[20]
Vrn-P7	*Vrn-B1b*	CCAATCTCACATGCCTCCAA	ATGCGCCATGAACAACAAAG	215 or 252	59	In this paper
Vrn-P8	*Vrn-D1a*	GTTGTCTGCCTCATCAAATCC	GGTCACTGGTGGTCTGTGC	1671	63	[20]
Vrn-P9	*vrn-D1*	GTTGTCTGCCTCATCAAATCC	AAATGAAAAGGAACGGAGCG	997	59	[20]
Vrn-P10	*Vrn-D1b*	GTTGTCTGCCTCATCAAATCC	AGGATGGCCAGGCCAAAACG	612 bp	60	[28]
Vrn-P11	*Vrn-D1b*	GTTGTCTGCCTCATCAAATCC	AGGATGGCCAGGCCAAAACT	612 bp	60	[28]
Vrn-P12	*vrn-B3*	ATGCTTTCGCTTGCCATCC	CTATCCCTACCGGCCATTAG	1140 or 2030	56	[9]
Vrn-P13	*Vrn-B3a*	CATAATGCCAAGCCGGTGAGTAC	ATGTCTGCCAATTAGCTAGC	1200	59	[9]
Vrn-P14	*Vrn-B3*c	GCTTTGAACTCCAAGGAGAA	ATAATCAGCAGGTGAACCAG	1401	52	In this paper
Vrn-P15	*vrn-B3/Vrn-B3*	ACTCATCATCACCACTTCCT	TAATGCTTAATTCGTGGCTG	1499	51	In this paper
Vrn-P16	*Vrn-B3* promoter	GTCCATACAAATCATGCCAC	TTCTGACAGTTTTAGTTGCG	491	51	In this paper
Vrn-P17	*Vrn-B3* promoter	GCTTTCGCTTGCCATCCCAT	GCGGGAACGCTAATCTCCTG	898	62	In this paper
Vrn-P18	*Vrn-B3* promoter	TTTGAGACAGGAGATTAGCG	ACCATCATGAGGCACCATTA	1131	53	In this paper
Vrn-P19	*Vrn-B3* promoter	GCTTTGAACTCCAAGGAGAA	ATAATCAGCAGGTGAACCAG	1425	52	In this paper
Vrn-P20	*Vrn-B3* promoter	CCGTTCACCATCTATTGCTC	CACCCAAATCCTTCATCTCA	1259	55	In this paper
Vrn-P21	CNV of *Vrn-A1*	CATTGTTCCTTCCTGTCCCACCC	ATTACTCGTACAGCCATCTCAGCC	1431	63	[29]
Vrn-B3-RT	-	GGAGGTGATGTGCTACGAGA	TTGTAGAGCTCGGCGAAGTC	147	55	In this paper
*β*-actin	-	GTTCCAATCTATGAGGGATACACGC	GAACCTCCACTGAGAACAACATTACC	422	56	[30]
Ppd-P1	*Ppd-D1a*	ACGCCTCCCACTACACTG	CACTGGTGGTAGCTGAGATT	288 or 2377	54	[15]
Ppd-P2	*Ppd-D1b*	ACGCCTCCCACTACACTG	GTTGGTTCAAACAGAGAGC	414 or 453	54	[15]
Ppd-P3	16 bp insertion Exon 8	GATGAACATGAAACGGG	GTCTAAATAGTAGGTACTAGG	320 or 336	52	[15]
Ppd-P4	TE deletion	AGGTCCTTACTCATACTCAATCTCA	CTCCCATTGTTGGTGTTGTTA	2612	50	[23]
Ppd-P5	2 kb deletion or TE insertion	CCATTCGAGGAGACGATTCAT	CTGAGAAAGAACAGAGTCAA	1005	55	[23]
Ppd-P6	5 bp deletion Exon 7	GAATGGCTTCTCCTGGTC	GATGGGCGAAACCTTATT	1,032 or 1,027	50	[23]
Ppd-P7	5 bp deletion Exon 7	GTGTCCTTTGCGAATCCTT	TTGGAGCCTTGCTTCATCT	184 or 179	53	[23]
Ppd-P8	Truncated Ppd-B1 gene in the ‘Chinese Spring’ allele	TAACTGCTCCTCACAAGTGC	CCGGAACCTGAGGATCATC	425	56	[31]
Ppd-P9	Intact Ppd-B1 copies in the ‘Chinese Spring’ allele	AAAACATTATGCATATAGCTTGTGTC	CAGACATGGACTCGGAACAC	994	58	[31]
Ppd-P10	Intact Ppd-B1 copies in the ‘Sonora64’/‘Timstein’ allele	CCAGGCGAGTGATTTACACA	GGGCACGTTAACACACCTTT	223	58	[31]

**Table 2:** Names and the molecular characterization of vernalization response alleles currently reported in polyploid wheat

**Locus**	**Allele**	**NCBI No.**	**Molecular characterization**	**Reference**
*Vrn-A1*	*vrn-A1*	AY747600	-	[20]
	*Vrn-A1a*	AY616458, AY616459	231-bp and 140-bp insertions at -439 bp and -348 bp, respectively.	[9]
	*Vrn-A1b*	AY616461	20-bp deletion at -157 bp	[9]
	*Vrn-A1c*	AY747599	5504-bp deletion at +1349 bp	[20]
	*Vrn-A1d*	AY616462	32-bp deletion at -214 bp	[9]
	*Vrn-A1e*	AY616463	54-bp deletion at -220 bp	[9]
*Vrn-B1*	*vrn-B1*	AY747604	-	[20]
	*Vrn-B1a*	AY747603	6850-bp deletion at +836 bp	[20]
	*Vrn-B1b*	FJ766015	6850-bp deletion at +836 bp and 37-bp deletion at +7992 bp	[22]
	*Vrn-B1c*	HQ593668, HQ130482	817-bp deletion and 0.4-kb duplication at +798 bp	[32]
				[25]
*Vrn-B3*	*vrn-B3*	DQ890162	-	[10]
	*Vrn-B3a*	DQ890165	5300-bp insertion at -592 bp	[10]
	*Vrn-B3b*	JN627519	890-bp insertion at -429 bp	In this paper
	*Vrn-B3c*	JQ082311	5300-bp insertion at -592 bp but 20-bp and 4-bp deletions at -3543 bp and -3591 bp	In this paper
*Vrn-D1*	*vrn-D1*	AY747606	-	[20]
	*Vrn-D1a*	AY747597	4235-bp deletion at +810 bp	[20]

When amplified with primer set Vrn-P12F/R (Table 1), we did not obtain any PCR product in the 2 winter cultivars Ji 874–109 and Hemai 26. Because primer set Vrn-P13F/R did not work very well in these2 cultivars, 5 primer sets (Vrn-P16 ~ Vrn-P20 in Table 1) were designed at the different positions of the *Vrn-B3* locus based on the sequence of DQ890165. All PCR products amplified in cultivars Ji 874–109 and Hemai 26 were then sequenced from both directions. Results indicated that sequences amplified with 4 primer sets were identical to *Vrn-B3a* (data not listed), whereas the sequence amplified with primer set Vrn-P19F/R (Table 1) identified 2 deletions in landrace cultivar Ji 874–109: a 20-bp fragment at -3543 bp and a 4-bp fragment at -3591 bp in the 5′-UTR (Table 2), when compared with the *Vrn-B3a* allele. This new *Vrn-B3* allele was designated *Vrn-B3c* allele (NCBI No: JQ082311) according to the nomenclature of Yan et al. [9], Fu et al. [20], and Shcherban et al. [25]. The cultivar Hemai 26 showed the same molecular characterization as that of *Vrn-B3a*.

### Expression of different *Vrn-B3* alleles in winter wheat and their association with days to heading (DH) and days to flowering (DF)

**Figure 3:**
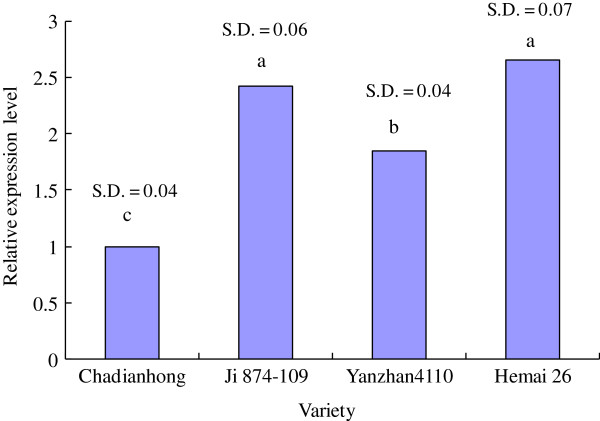
**Comparison of relative expression levels of 4 varieties with different *Vrn-B3 *** **alleles by real-time PCR.**

Four cultivars, i.e., Ji 874–109 (*Vrn-B3c*), Yanzhan 4110 (*vrn-B3*), Chadianhong (*Vrn-B3b*), and Hemai 26 (*Vrn-B3a*), with different *Vrn-B3* alleles were selected to compare expression levels by real-time PCR. Sequencing the *Vrn-B3* coding region (amplification with primer set Vrn-P15 R/F in Table 1) of the 4 above-mentioned cultivars showed that they were 100% identical on the DNA level (data not shown). Real-time PCR (Figure 3) indicated the landrace cultivar Chadianhong with the *Vrn-B3b* allele possessed significantly lower expression level than Yanzhan 4110 with the recessive *vrn-B3* allele, suggesting that the 890-bp insertion in *Vrn-B3b* allele possibly contributed to its reduced expression level. However, wheat accession Ji 874–109 with the *Vrn-B3c* allele did not show significant difference at the transcript level from Hemai 26 with the *Vrn-B3a* allele, even though the expression level of the *Vrn-B3c* allele was relatively lower than that of the *Vrn-B3a* allele. This suggested that the 2 deletions (20-bp and 4-bp) may not have had a significant effect on *Vrn-B3a* promoter activity. In addition, RT-PCR indicated that Hemai 26 possessed the highest expression at the transcript level of *Vrn-B3* alleles among the 4 cultivars. Although the difference in *Vrn-B3* expression between Hemai 26 and Ji 874–109 was not significant on the transcript level (Figure 3), these results suggested that the 5300-bp insertion in *Vrn-B3a* and *Vrn-B3c* alleles possibly contributeed to their increased transcription.

Currently, the Chinese cultivar Yanzhan 4110 is being used as a control for releasing new wheat cultivars in yield comparison trials of Winter Wheat Regional Field Testing of the Yellow and Huai Valley southern region. Under the vernalization conditions in the field and compared with Yanzhan 4110 (192 DH and 196 DF), wheat cultivar Chadianhong (202 DH and 207 DF) with the *Vrn-B3b* allele headed 10 days later and flowered 11 days later, and Hemai 26 (197 DH and 202 DF) with the *Vrn-B3a* allele headed 5 days later and flowered 6 days later. The cultivar Ji 874–109 (191 DH and 197 DF) with the *Vrn-B3c* allele, however, showed only ± 1 day differences in heading and flowering times from those of Yanzhan 4110 (see Additional file 1).

Moreover, the 4 above-mentioned cultivars without vernalization were further investigated for their heading and flowering days, in a greenhouse with 16 h daylight cycles, to test their vernalization requirements. Results indicated that 3 cultivars, i.e., Ji 874–109 (89 DH and 101 DF), Hemai 26 (138 DH and 149 DF), and Chadianhong (158 DH and 169 DF) headed 9, 58, and 78 days later and flowered 9, 57, and 77 days later, respectively, when compared with Yanzhan 4110 (80 DH and 92 DF).

### Distribution of vernalization response and *Ppd-D1* alleles in Chinese winter wheat cultivars surveyed

Based on sequences of AY747600 and AY747601, we developed a new co-dominant marker Vrn-P2F/R (Table 1) for more precise identification of the *Vrn-A1b* allele (Figure 1B) by replacing the marker Vrn-P1F/R previously developed by Yan et al. [9]. Sequencing results showed reliability of the new marker Vrn-P2F/R. Using the allele-specific primer sets Vrn-P1F/R ~ Vrn-P4F/R, we showed that 161 out of 173 winter wheat cultivars possessed the recessive *vrn-A1* allele at the *Vrn-A1* locus. Nine of these possessed the *Vrn-Ala* allele, and 2 cultivars (Yumai 1 and Wuyimai) possessed the *Vrn-Alb* allele (Table 3). However, a cultivar (Hemai 26) from Henan did not show any PCR product when amplified with the above-mentioned 4 primer sets, possibly indicating that there was a large deletion at the *Vrn-A1* locus of Hemai 26. Therefore, the above-mentioned later heading and flowering times of Hemai 26 may be primarily because of its *Vrn-A1* deletion, even though the expression level of the *Vrn-B3* allele in this cultivar was not significantly lower than that of Yanzhan 4110. Genotyping results suggested that the recessive allele *vrn-A1* was predominant at the *Vrn-A1* locus in winter wheat cultivars from the Yellow and Huai Valley of China. Of the 51 spring wheat we surveyed, 31, 18, and 2 had *vrn-A*, *Vrn-A1a*, and *Vrn-V1b* alleles, respectively (see Additional file 1).

**Table 3:** **Distribution of known vernalization response alleles and *Ppd-D1 *** **haplotypes in bread wheat cultivars from the Yellow and Huai Valley of China**

**Locus**	**Allele/haplotype**	**Henan**	**Hebei**	**Shaanxi**	**Shandong**	**Shanxi**	**Others**	**Total**
**Sample No.**		**85**	**20**	**19**	**13**	**7**	**29**	**173**
*Vrn-A1*	*vrn-A1*	79	20	19	13	7	23	161
	*Vrn-A1a*	4	0	0	0	0	5	9
	*Vrn-A1b*	1	0	0	0	0	1	2
*Vrn-B1*	*vrn-B1*	80	19	18	12	7	25	161
	*Vrn-B1a*	5	1	1	1	0	4	12
*Vrn-B3*	*vrn-B3*	84	18	19	13	7	29	170
	*Vrn-B3a*	1	0	0	0	0	0	1
	*Vrn-B3b*	0	1	0	0	0	0	1
	*Vrn-B3c*	0	1	0	0	0	0	1
*Vrn-D1*	*vrn-D1*	54	13	9	8	6	17	107
	*Vrn-D1a*	13	2	3	2		4	24
	*Vrn-D1b*	18	5	7	3	1	8	42
*Ppd-D1*	*Hapl-I*	84	15	19	12	6	19	155
	*Hapl-II*	0	2	0	0	0	1	3
	*Hapl-III*	0	0	0	0	0	1	1
	*Hapl-IV*	1	0	0	1	1	5	8
	*Hapl-VII*	0	2	0	0	0	2	4
	*Hapl-VIII*	0	0	1	0	0	1	2

Two previously developed primer sets Vrn-P5F/R and Vrn-P6F/R were used to identify recessive and dominant alleles at the *Vrn-B1* locus in Chinese winter wheat cultivars. In order to identify *Vrn-B1a* and *Vrn-B1b* alleles, recently reported by Santra et al. [22] and Milec et al. [32], we developed a new co-dominant marker Vrn-P7F/R (Table 1) with a 215-bp fragment for the *Vrn-B1a* allele and a 252-bp fragment for the *Vrn-B1b* allele (Figure 1C), based on the characterization of *Vrn-B1* alleles (Figure 2B). Sequencing results confirmed its reliability. Due to the absence of the *Vrn-B1b* allele in the Chinese winter wheat cultivars surveyed in this study, we also collected 51 currently popular spring wheat cultivars from Gansu for detection of the *Vrn-B1b* allele, using the co-dominance of primer set Vrn-P7F/R (Figure 1C). Genotyping results using primer sets Vrn-P5F/R, Vrn-P6F/R, and Vrn-P7F/R (Table 1) indicated that 161 out of 173 winter wheat cultivars had the recessive *vrn-B1* allele (accounting for 93.1%), while 12 cultivars had the *Vrn-B1a* allele (accounting for 6.9%; Table 3). This suggested that the recessive allele *vrn-B1* was predominant at the *Vrn-B1* locus in winter wheat cultivars from the Yellow and Huai Valley of China. Of the 51 spring wheat cultivars we surveyed, 13, 24, and 14 had *vrn-B1*, *Vrn-B1a*, and *Vrn-B1b* alleles, respectively (see Additional file 1).

Among the Chinese winter wheat cultivars we surveyed, 170 out of 173 cultivars had the recessive *vrn-B3* allele, accounting for 98.3%; while only 1 cultivar (Hemai 26) was identified as having *Vrn-B3a*, 1 cultivar (Chadianhong) as having *Vrn-B3b*, and 1 cultivar (Ji 874–109) as having *Vrn-B3c*, as described above (Table 3). This suggested that the recessive *vrn-B3* allele was the predominant genotype at the *Vrn-B3* locus in winter wheat cultivars from the Yellow and Huai Valley of China. In the spring wheat we surveyed, all 51 cultivars have the same allele *vrn-B3*.

Genotyping results with primer sets Vrn-P8F/R and Vrn-P9F/R showed that 107 out of 173 winter wheat cultivars possessed the recessive *vrn-D1* allele (accounting for 61.9%), and 66 cultivars possessed the dominant *Vrn-D1* allele (Table 3). Further identification with markers Vrn-P10F/R and Vrn-11 F/R showed that 24 and 42 out of 66 cultivars had *Vrn-D1a* (accounting for 13.9%) and *Vrn-D1b* alleles (accounting for 24.2%), respectively. These data suggested that the dominant *Vrn-D1a* and *Vrn-D1b* alleles were prevalent in Chinese winter wheat cultivars even though the recessive *vrn-D1* allele was still the predominant genotype at the *Vrn-D1* locus in winter wheat cultivars from the Yellow and Huai Valley of China. A similar observation was described by Zhang et al. [24,28]. Of the 51 spring wheat cultivars we surveyed, 32 and 19 had *vrn-D1* and *Vrn-D1a* alleles, respectively (see Additional file 1).

Characterization of the allelic combination of vernalization response genes at *Vrn-A1*, *Vrn-B1*, *Vrn-D1*, and *Vrn-B3* loci revealed that 92 out of the 173 Chinese winter wheat cultivars surveyed had *vrn-A1/vrn-B1/vrn-D1/vrn-B3* (53.2%), 30 cultivars possessed *vrn-A1/vrn-B1/Vrn-D1a/vrn-B3* (17.3%), 37 cultivars possessed *vrn-A1/vrn-B1/Vrn-D1b/vrn-B3* (21.4%), 6 cultivars possessed *vrn-A1/Vrn-B1a/vrn-D1/vrn-B3*, 5 cultivars possessed *Vrn-A1a/vrn-B1/vrn-D1/vrn-B3*, 2 cultivars (Huixianhong and Fan 6) possessed *vrn-A1/Vrn-B1a/Vrn-D1a/vrn-B3*, 2 cultivars (Xibeiaigezi and Aimengniu) possessed *vrn-A1/Vrn-B1a/Vrn-D1b/vrn-B3*, 2 cultivars (Xichuan 76–9 and Zhenghua 3563) possessed *Vrn-A1a/vrn-B1/Vrn-D1a/vrn-B3*, 2 cultivars (Zhenghua 0840–3 and Xinkehan 9) possessed *Vrn-A1a/vrn-B1/Vrn-D1b/vrn-B3*, 1 cultivar (Ji 874–109) possessed *vrn-A1/Vrn-B1a/vrn-D1/Vrn-B3c*, 1 cultivar (Chadianhong) had *vrn-A1/vrn-B1/Vrn-D1b/Vrn-B3b*, 1 cultivar (Hemai 26) possessed *vrn-B1/vrn-D1/Vrn-B3a* (possible lack of the *Vrn-A1* gene), 1 cultivar (Yumai 1) possessed *Vrn-A1b/vrn-B1/vrn-D1/vrn-B3* and 1 cultivar Wuyimai possessed *Vrn-A1b/Vrn-B1a/vrn-D1/vrn-B3*. A total of 14 allelic combinations for vernalization response genes were discovered in the Chinese winter wheat surveyed. All these suggested that the recessive allelic combination of *vrn-A1/vrn-B1/vrn-D1/vrn-B3* was predominant, but the combinations of *vrn-A1/vrn-B1/Vrn-D1a/vrn-B3* and *vrn-A1/vrn-B1/Vrn-D1b/vrn-B3* were prevalent in winter wheat cultivars from the Yellow and Huai Valley of China.

### Distribution of the *Ppd-D1* gene in Chinese winter wheat cultivars

A series of molecular markers (Ppd-P1 ~ Ppd-P7, Table 1) were used to identify *Ppd-D1* sequence polymorphisms according to the methods described by Guo et al. [23]. In total, 3 polymorphisms were found in bread wheat cultivars, i.e. a 2089-bp deletion in exon 8, the *mariner*-like transposable element (TE) insertion in intron 1, and a 5-bp deletion in exon 7; two polymorphisms were not found, i.e., a 16-bp insertion in exon 8 and a 24-bp plus a 15-bp insertions in the 2-kp upstream region.

In the 173 winter wheat cultivars surveyed in this study, 161 cultivars had a 2089-bp deletion upstream of the coding region and possessed the photoperiod-insensitive *Ppd-D1a* allele, which causes early flowering, whereas the remaining 12 cultivars had the photoperiod-sensitive *Ppd-D1b* allele, which causes later flowering. Moreover, 5 cultivars were identified as possessing the *mariner*-like TE in intron 1 of the *Ppd-D1* gene, and 10 cultivars were identified as possessing 5-bp deletion in exon 7, which created a frame shift and resulted in a nonfunctional protein. All 173 cultivars possessed a 16-bp deletion containing the last 2 bases of the CCT domain in exon 8. However, no cultivar was found to have 24-bp plus 15-bp insertions in the 2-kb upstream region reported in synthetic wheats and *A. tauschii* accessions by Guo et al. [23].

Furthermore, 6 combinations of the 3 above-mentioned polymorphisms (Table 4) were examined in winter wheat cultivars from the Yellow and Huai Valley of China. However, there were 2 novel *Ppd-D1* polymorphism combinations identified in this study when compared to those reported by Guo et al. [23], i.e., absence of 2089 bp in exon 8 and presence of both TE in intron 1 and 5 bp in exon 7 for 4 cultivars: Danmai 1 introduced from Danmark (213 DH and 217 DF), Ji 923235 (194 DH and 198 DF), Ji 874–109 (191 DH and 197 DF), and Huapeiai (191 DH and 197 DF), and absence of 2089 bp in exon 8, TE in intron 1, and 5 bp in exon 7 for 2 cultivars Damuzhiai (210 DH and 215 DF) and Xian 83(104)-11 Zhong “S” (202 DH and 208 DF). We designated them as *Hapl-VII* (absence of 2089 bp in exon 8 and presence of both TE in intron 1 and 5 bp in exon 7) and *Hapl-VIII* (absence of 2089 bp in exon 8, TE in intron 1 and 5 bp in exon 7) according to the nomenclature of Guo et al. [23]. Of 6 polymorphism combinations, *Hapl-I* was the most prevalent, found in 89.6% of Chinese winter wheat cultivars surveyed from the Yellow and Huai Valley of China (Table 3).

**Table 4:** ***Ppd-D1 *** **haplotypes identified in bread wheat cultivars from the Yellow and Huai Valley of China**

***Ppd-D1 *** **Haplotype**	**Sample no.**	**24 bp plus 15 bp**	**2 kb**	**TE**	**5 bp**	**16 bp**
I	155	-	-	-	+	-
II	3	-	+	-	+	-
III	1	-	+	+	+	-
IV	8	-	+	-	-	-
V	0	-	+	-	+	+
VI	0	+	+	-	+	+
VII	4	-	-	+	+	-
XIII	2	-	-	-	-	-

Insertions and deletions are indicated by + and -, respectively.

### Copy number variations (CNVs) at the *Ppd-B1* and *Vrn-A1* loci

CNV is a type of mutation that has been widely studied in human genetics; these studies have sharply increased in plants recently due to its possible influence on phenotype. Based on the report of Díaz et al. [31], the *Ppd-B1a* gene has 3 types in view of CNV, i.e., truncated ‘Chinese Spring’ *Ppd-B1a* allele, intact ‘Chinese Spring’ *Ppd-B1a* allele, and ‘Sonora64’ allele. Wheat plants with the ‘Sonora64’ *Ppd-B1a* allele have been shown to flower earlier than those with the ‘Chinese Spring’ *Ppd-B1a* allele [31]. Identification of the above 3 *Ppd-B1a* alleles by specific primers (Ppd-P8 ~ Ppd-P10, Table 1) indicated that 109, 42, and 51 wheat cultivars possessed the truncated ‘Chinese Spring’ *Ppd-B1a*, intact ‘Chinese Spring’ *Ppd-B1a* and ‘Sonora64’ alleles, respectively, in the surveyed winter wheat from the Yellow and Huai Valley of China (see Additional file 1). Interestingly, all 42 cultivars with the intact ‘Chinese Spring’ *Ppd-B1a* allele contained the truncated ‘Chinese Spring’ *Ppd-B1a* allele, and 27 out of 51 cultivars with the intact ‘Sonora64’ allele contained the truncated ‘Chinese Spring’ *Ppd-B1a* allele.

CNV of the *Vrn-A1* gene were examined by the marker Pri_21F/R previously developed by Eagles et al. [29]. Because PCR products amplified by this marker contained more than one fragment and could not be directly sequenced even though we tried several times, 34 out of 173 winter wheat cultivars were selected to identify CNV of *Vrn-A1* by sequencing sub-clones. Results indicated that 18 wheat cultivars possessed both C and T sequences in exon 4 and the other 16 remaining cultivars only possessed the C sequence in exon 4 of the *Vrn-A1* gene (see Additional file 1).

## Discussion

In the Yellow and Huai wheat production region of China, the grain-filling stage of winter wheat cultivars usually lasts approximate 40 days from late April to early June for almost every cropping seasons; this may fluctuate according to the environmental characteristics of specific regions or provinces. However, farmers generally would like to plant early-maturing wheat cultivars in their fields due to the frequent presence of dry-hot winds in early June or late May, especially in Henan, which is the most important wheat production province in view of the yield and area in China. Based on a number of studies [8,15,20,31], vernalization and photoperiod response genes as well as their copy number variations have an important influence on plant flowering and maturity. In this study, we found that certain combinations of vernalization alleles and photoperiod haplotypes showed early-maturing characteristics, which would possibly provide useful information for screening early-maturing wheat cultivars by marker-assisted selection (MAS). For example, the winter wheat cultivars surveyed with the combination of *vrn-A1/Vrn-B1b/Vrn-B3a/Vrn-D1a/Ppd-D1a* headed and flowered an average of 3 days earlier than cultivars with the combination of *vrn-A1/vrn-B1/Vrn-B3a/Vrn-D1a/Ppd-D1a*. However, we did not exactly compare them due to the small number of cultivars with the exact same vernalization response and photoperiod alleles for certain combinations.

Since the vernalization response gene was cloned ten years ago [6,8,33,34], a number of functional molecular markers, derived from polymorphic sites within genes that directly affect phenotypic trait variation, have been developed for identification of diverse vernalization response alleles in ployploid wheat [8,9,20,22,25,32]. Also, several vernalization alleles have been identified in bread and durum wheat cultivars from different geographical environments. Even though some similar studies have been conducted in Chinese winter wheat cultivars from different agro-ecological zones, the large scale genotyping of wheat germplasms has still not been performed to provide details on vernalization and photoperiod response genes in winter wheat cultivars from the Yellow and Huai Valley of China, especially in landrace cultivars and more recent cultivars, because in this wheat region more than 50 new wheat cultivars are released every year, and more than 3000 landrace and historical cultivars are kept in different wheat germplasm banks. Therefore, further screening of the relatively superior genotypes of vernalization response and photoperiod genes would be beneficial for improving the adaptability of bread wheat.

Photoperiod response genes play an import role in wheat growth and development. Photoperiod insensitive cultivars flower under short-day and long-day conditions, whereas photoperiod-sensitive cultivars usually delay heading and flowering and may even not undergo heading if the day length and number of long days do not meet the minimum requirement of sensitive cultivars to head and flower. However, few studies have focused on identification of allelic variation of photoperiod genes in bread wheat and only 2 alleles have been found at each locus, i.e., photoperiod insensitive (*Ppd-D1a* and *Ppd-B1a*) and photoperiod sensitive (*Ppd-D1b* and *Ppd-B1b*). Moreover, 5 *Ppd-D1* alleles were identified by Beales et al. [15], but only the 2 kb deletion was associated with photoperiod insensitivity. However, those five alleles were expressed as haplotypes by Guo et al. [23] due to the simultaneous presence of more than one polymorphism in the *Ppd-D1* locus. Therefore, we suggest using the method of designating haplotypes to name the different combinations of diverse polymorphisms, instead of alleles, in describing photoperiod gene variations in the D genome of bread wheat cultivars. Based on our study, it seems that not only a 2-kb deletion, but also other polymorphisms have an impact on days to heading and flowering due to the obvious differences between Chinese cultivars with *HapVII* and *Hap VIII*. However, more work needs to be conducted to further support this conclusion.

Changes in the promoter sequence are quite common in the known vernalization response genes in bread wheat. Four of 5 *Vrn-A1* alleles previously reported were resulted from mutations of the promoter sequence at the *Vrn-A1* locus. In this study, 2 new alleles, *Vrn-B3b* and *Vrn-B3c*, were caused by changes in the promoter sequence at the *Vrn-B3* locus. A 5300-bp insertion in the promoter region of *Vrn-B3* has been shown to cause early flowering [10]. However, in this study, the new allele *Vrn-B3b*, having an 890-bp insertion significantly reduced the expression level of the gene and caused later heading and flowering. Our results also suggested that the special promoter sequence of *Vrn-B3b* may be useful to further analysis of promoter influence on the expression levels of genes.

A previous study indicated that overexpression of the *Vrn-3* gene in winter wheat could result in up-regulation of the *Vrn-1* gene and a spring growth habit [35]. We also found that the wheat cultivar with the *Vrn-B3a* allele possessed significantly higher expression at the transcript level, as shown by RT-PCR. Therefore, it is plausible that the *Vrn-B3a* allele in wheat cultivar Hope caused FT overexpression and early flowering [10]. However, in this study, the wheat cultivar Hemai 26 with the *Vrn-B3a* allele also exhibited deletion of *Vrn-A1*, and its later flowering was possibly caused by this *Vrn-A1* deletion because the *Vrn-A1* gene may have a greater impact on flowering time than the *Vrn-B3* gene, even though Hemai 26 exhibited overexpression of *Vrn-B3*. However, Chadianhong, which possessed the *Vrn-B3b* allele, exhibited later flowering possibly because of its 890-bp insertion, which reduced expression of the *Vrn-3* gene, instead of a 5300-bp insertion which would result in increased expression. Therefore, the later heading and flowering times of some cultivars surveyed in this study may have resulted from the relatively low expression of *Vrn-B3*, leading to the reduced expression of the *Vrn-1* gene. However, due to the discovery of the *Vrn-4* gene, there are still other unknown genes related to vernalization and photoperiod response in bread wheat, and the differences amongst days to heading and flowering of diverse cultivars should result from the combination of multiple determinants including vernalization, photoperiod response genes and their CNVs.

## Conclusion

We characterized the allelic variations of vernalization and photoperiod response genes as well as their CNVs in 173 winter wheat and 51 spring wheat cultivars of China, found 2 new *Vrn-B3* alleles, 1 new *Vrn-A1* allele and 2 new polymorphism combinations at the photoperiod locus, and developed functional markers for identifying different vernalization response alleles. Cultivars with new *Vrn-B1* or *Vrn-A1* alleles headed and flowered significantly later under the condition of non-vernalization. This study could provide useful information for screening relatively superior wheat cultivars for better adaptability and maturity in Yellow and Huai Valley of China.

## Methods

### Plant materials

In this study, 173 winter wheat accessions composed of landrace cultivars and current popular cultivars from the Yellow and Huai Valley of China, mainly collected from Henan, Hebei, Shaanxi, Shanxi and Shandong, were planted in October, 2010 and harvested in the June, 2011 cropping season at the Zhengzhou Scientific Research and Education Center of Henan Agricultural University (longitude: 113.6; latitude: 34.9) under local management practices. The field experiment was performed using a completely random design. Each plot contained four 200 cm-long rows with 23 cm between neighboring rows and 10 cm between neighboring plants. All surveyed cultivars, vernalized through the winter with an average temperature of 1.3°C (December, January, and February) in 2011, grew very well with the supporting net and no lodging was present in the trial. DH and DF were investigated for each cultivar surveyed before harvesting.

In addition, 51 spring wheat cultivars, kindly provided by Liu Baolong from Northwest Institute of Plateau Biology of Chinese Academy of Sciences, were used to detect the vernalization response alleles at *Vrn-A1*, *Vrn-B1* and *Vrn-D1* loci.

### PCR amplification

The genomic DNA of each cultivar surveyed was individually extracted from 3 pulverized kernels, following a method described by Chen et al. [36]. The PCR amplification reactions were conducted in a 25-μL reaction volume containing 100 ng genomic DNA, 10 pmol of each primer (Table 1), 200 μM of each dNTP, 1× *Taq* DNA polymerase reaction buffer with 1.5 μM MgCl_2_, and 0.5 unit of *Taq* DNA polymerase using PTC-200 Peltier Thermocycler or ABI 9700. The cycling conditions were as follows: 94°C for 5 min followed by 35 cycles of 94°C for 40 s, 50°C to 65°C for 40 s (primer-specific annealing temperatures, see Table 1), and 72°C for 1.5 min, followed by a final 10-min extension at 72°C. PCR products were separated by electrophoresis either on a 1.5% ~ 2.5% agarose gel stained with ethidium bromide and visualized using UV light, or on a 6% polyacrylamide gel and resolved by silver staining [37].

### DNA sequencing

PCR products were purified using Quick DNA Extraction Kit (Takara, Otsu, Japan), ligated into the pGEM-T Easy vector, and transformed into competent cells of the *Escherichia coli* DH-5*α* strain. Plasmids with targeted fragments detected by colony PCR were extracted using a Plasmid Rapid Isolation Kit (Biodev-tech Company, Beijing, China). Five clones for each new allele were sequenced from both strands by SinoGenoMax Co., Ltd (Beijing, China).

Multiple alignments of sequences and translations of nucleotide sequences into amino acid sequences were performed by DNAMAN Version 6.0 software. Graphical data of sequencing results were analyzed by Chromas Version 1.4.5.

### Real-time quantitative reverse transcription PCR

A total RNA of 4 wheat cultivars with different *Vrn-B3* alleles were extracted from 2-month-old seedlings according to the method of Chen et al. [30]. DNA was removed by digestion with DNAse I (Qiagen, China) before reverse transcription. First-stand cDNA was synthesized using M-MLV transcriptase (Invitrogen, Carlsbad, CA, USA). Coding regions of different *Vrn-B3* alleles in cDNA were sequenced with the Vrn-P15F/R primer set (Table 1) in order to confirm no sequence change in coding regions of the 4 *Vrn-B3* alleles surveyed. The primer set Vrn-B3-RT_F/R (Table 1) was designed by Software Primer Premier 5.0 for RT-PCR amplification. Amplification with *β*-actin primers (Table 1) was used as an internal control to normalize all data. The relative quantification method (2^-ΔΔ*C*^_T_) was used to evaluate quantitative variation between the 3 replicates, following the method of Livak and Schmittgen [38].

## Abbreviations

Vrn: Vernalization; Ppd: Photoperiod; CNV: Copy number variation; PCR: Polymerase chain reaction; TE: Transposable element; CCT: CONSTANS, CO-like, and TOC1; UTR: Un-translate region; DH: Days to heading; DF: Days to flowering.

## Competing interests

The authors declare that they have no competing interests.

## Authors’ contributions

FC and DC designed and prepared the manuscript. FC, GX and JZ performed identification of phenotypes and genotypes in winter wheat cultivars surveyed. AZ and XS participated in identification of genotypes in spring wheat cultivars. All authors read and approved the final manuscript.

## Supplementary Material

Additional file 1 Origin, growth habit, flowering and heading days, allelic variations of vernalization and photoperiod response genes, and copy number variations of Chinese wheat cultivars surveyed. Click here for file

## References

[B1] Slafer GA Genetic basis of yield as viewed from a crop physiologist’s perspective Ann Appl Biol 2003 13 117 128 10.1111/j.1744-7348.2003.tb00237.x

[B2] Barrett B Bayram M Kidwell K Identifying AFLP and microsatellite markers for vernalization response gene Vrn-B1 in hexaploid wheat (Triticum aestivum L.) using reciprocal mapping populations Plant Breed 2002 13 400 406 10.1046/j.1439-0523.2002.732319.x

[B3] Dubcovsky J Lijavetzky D Appendino L Tranquilli G Comparative RFLP mapping of Triticum monococcum genes controlling vernalization requirement Theor Appl Genet 1998 13 968 975 10.1007/s001220050978

[B4] Galiba G Quarrie SA Sutka J Morgounov A Snape JW RFLP mapping of the vernalization (Vrn1) and frost resistance (Fr1) genes on chromosome 5A of wheat Theor Appl Genet 1995 13 1174 1179 24173081 10.1007/BF00222940

[B5] Iwaki K Nishida J Yanagisawa T Yoshida H Kato K Genetic analysis of Vrn-B1 for vernalization requirement by using linked dCAPS markers in bread wheat ( Triticum aestivum L.) Theor Appl Genet 2002 13 571 576 10.1007/s00122-001-0769-0 12582660

[B6] Trevaskis B Bagnall DJ Ellis MH Peacock WJ Dennis ES MADS box genes control vernalization-induced flowering in cereals Proc Natl Acad Sci U S A 2003 13 13099 13104 10.1073/pnas.1635053100 14557548 PMC240751

[B7] Preston JC Kellogg EA Discrete developmental roles for temperate cereal grass VERNALIZATION1/FRUITFULL-Like genes in flowering competency and the transition to flowering Plant Physiol 2008 13 265 276 18024551 10.1104/pp.107.109561 PMC2230560

[B8] Yan L Loukoianov A Tranquilli G Helguera M Fahima T Dubcovsky J Positional cloning of the wheat vernalization gene VRN1 Proc Natl Acad Sci U S A 2003 13 6263 6268 10.1073/pnas.0937399100 12730378 PMC156360

[B9] Yan L Loukoianov A Blechl A Tranquilli G Ramakrishna W SanMiguel P Bennetzen JL Echenique V Dubcovsky J The wheat VRN2 gene is a flowering repressor down-regulated by vernalization Science 2004 13 1640 1644 10.1126/science.1094305 15016992 PMC4737501

[B10] Yan L Fu D Lin C Blechl A Tranquilli G Bonafede M Sanchez A Valarik M Dubcovsky J The wheat and barley vernalization gene VRN3 is an orthologue of FT Proc Natl Acad Sci U S A 2006 13 19581 19586 10.1073/pnas.0607142103 17158798 PMC1748268

[B11] Trevaskis B Hemming MN Peacock WJ Dennis ES HvVRN2 responds to daylength, whereas HvVRN1 is regulated by vernalization and developmental status Plant Physiol 2006 13 1397 1405 10.1104/pp.105.073486 16500994 PMC1435809

[B12] Faure S Higgins J Turner A Laurie DA The flowering locus T-like gene family in barley (Hordeum vulgare) Genetics 2007 13 599 609 10.1534/genetics.106.069500 17339225 PMC1893030

[B13] Worland AJ Snape JW Bonjean AP, Angus WP Genetic basis of worldwide wheat varietal improvement World Wheat Book – A History of Wheat Breeding 2001 Paris, France: Lavoisier Publishing 59 100

[B14] Welsh JR Keim DL Pirasteh B Richards RD Sears ER, Sears LMS Genetic control of photoperiod response in wheat Proceedings of the 4th International Wheat Genetic Symposium 1973 Columbia, MO, USA: University of Missouri Press 879 884

[B15] Beales J Turner A Griffiths S Snape JW Laurie DA A Pseudo-Response Regulator is misexpressed in the photoperiod insensitive *Ppd-D1a* mutant of wheat (*Triticum aestivum* L.) Theor Appl Genet 2007 13 721 733 10.1007/s00122-007-0603-4 17634915

[B16] Wilhelm EP Turner AS Laurie DA Photoperiod insensitive Ppd-A1a mutations in tetraploid wheat (Triticum durum Desf.) Theor Appl Genet 2009 13 285 294 10.1007/s00122-008-0898-9 18839130

[B17] Scarth R Law CN The control of day-length response in wheat by the group 2 chromosomes Z Pflanzenzüchtung 1984 13 140 150

[B18] Worland AJ Börner A Korzun V Li WM Petrovíc S Sayers EJ The influence of photoperiod genes on the adaptability of European winter wheats Euphytica 1998 13 385 394 10.1023/A:1018327700985

[B19] Tanio M Kato K Development of near-isogenic lines for photoperiod-insensitive genes, Ppd-B1 and Ppd-D1, carried by the Japanese wheat cultivars and their effect on apical development Breed Sci 2007 13 65 72 10.1270/jsbbs.57.65

[B20] Fu DL Szucs P Yan LL Helguera M Skinner JS von Zitzewitz J Hayes PM Dubcovsky J Large deletions within the first intron in VRN-1 are associated with spring growth habit in barley and wheat Mol Genet Genom 2005 13 54 65 10.1007/s00438-004-1095-4 15690172

[B21] Iqbal MA Navabi RC Yang DF Salmon DF Spaner D Molecular characterization of vernalization response genes in Canadian spring wheat Genome 2007 13 511 516 10.1139/G07-028 17612620

[B22] Santra DK Santra M Allan RE Campbell KG Kidwell KK Genetic and molecular characterization of vernalization genes Vrn-A1, Vrn-B1, and Vrn-D1 in spring wheat germplasm from the Pacific Northwest region of the U.S.A Plant Breed 2009 13 576 584

[B23] Guo ZA Song YX Zhou R Ren ZL Jia JZ Discovery, evaluation and distribution of haplotypes of the wheat Ppd-D1 gene New Phytol 2010 13 841 851 10.1111/j.1469-8137.2009.03099.x 20002313

[B24] Zhang XK Xia XC Xiao YG Dubcovsky J He ZH Allelic variation at the vernalization genes Vrn-A1, Vrn-B1, Vrn-D1 and Vrn-B3 in Chinese common wheat cultivars and their association with growth habit Crop Sci 2008 13 458 470 10.2135/cropsci2007.06.0355

[B25] Shcherban AB Efremova TT Salina EA Identification of a new Vrn-B1 allele using two near-isogenic wheat lines with difference in heading time Mol Breeding 2012 13 675 685 10.1007/s11032-011-9581-y

[B26] Golovnina K Kondratenko EY Blinov AG Goncharov NP Molecular characterization of vernalization loci VRN-1 in wild and cultivated wheats BMC Plant Biol 2010 13 168 10.1186/1471-2229-10-168 20699006 PMC3095301

[B27] He ZH Rajaram S Xin ZY Huang GZ A history of wheat breeding in China 2001 Mexico, DF: CIMMYT 1 94

[B28] Zhang J Wang Y Wu S Yang J Liu H Zhou Y A single nucleotide polymorphism at the Vrn-D1 promoter region in common wheat is associated with vernalization response Theor Appl Genet 2012 13 1697 1704 10.1007/s00122-012-1946-z 22875177

[B29] Eagles HA Cane K Trevaskis B Veery wheats carry an allele of Vrn-A1 that has implications for freezing tolerance in winter wheats Plant Breed 2011 13 413 418 10.1111/j.1439-0523.2011.01856.x

[B30] Chen F Zhang FY Li HH Morris CF Cao YY Shang XL Cui DQ Allelic variation and distribution independence of Puroindoline b-B2 variants and their association with grain texture in wheat Mol Breed 2013 13 399 409 10.1007/s11032-013-9879-z

[B31] Díaz A Zikhali M Turner AS Isaac P Laurie DA Copy number variation affecting the photoperiod-B1 and vernalization-A1 genes is associated with altered flowering time in wheat (Triticum aestivum) PLoS ONE 2012 13 e33234 doi: 10.1371/journal.pone.0033234 10.1371/journal.pone.0033234 22457747 PMC3310869

[B32] Milec Z Tomková L Sumíková T Pánková K A new multiplex PCR test for the determination of Vrn-B1 alleles in bread wheat (*Triticum aestivum* L.) Mol Breeding 2012 doi:10.1007/s11032-011-9621-7

[B33] Danyluk J Kane NA Breton G Limin AE Fowler DB Sarhan F TaVRT-1, a putative transcription factor associated with vegetative to reproductive transition in cereals Plant Physiol 2003 13 1849 1860 10.1104/pp.103.023523 12913142 PMC181271

[B34] Murai K Miyamae M Kato H Takumi S Ogihara Y WAP1: a wheat APETALA1 homolog, plays a central role in the phase transition from vegetative to reproductive growth Plant Cell Physiol 2003 13 1255 1265 10.1093/pcp/pcg171 14701921

[B35] Li CX Dubcovsky J Wheat FT protein regulates VRN1 transcription through interactions with FDL2 Plant J 2008 13 543 554 10.1111/j.1365-313X.2008.03526.x 18433437 PMC4739743

[B36] Chen F Xu HX Zhang FY Xia XC He ZH Wang DW Dong ZD Zhan KH Cheng XY Cui DQ Physical mapping of puroindoline b-2 genes and molecular characterization of a novel variant in durum wheat (Triticum turgidum L.) Mol Breed 2011 13 153 161 10.1007/s11032-010-9469-2

[B37] Bassam BJ Caetano-Anollés G Gresshoff PM Fast and sensitive silver staining of DNA in polyacrylamide gels Anal Biochem 1991 13 80 83 10.1016/0003-2697(91)90120-I 1716076

[B38] Livak KJ Schmittgen TD Analysis of relative gene expression data using real-time quantitative PCR and the 2 ^-∆∆C^_T_ method Methods 2001 13 402 408 10.1006/meth.2001.1262 11846609

